# Supplementary figures and images for: DNA methylation mediates the effect of cocaine use on HIV severity
Source: Clin Epigenetics. 2020 Sep 14;12:140. doi: 10.1186/s13148-020-00934-1 (PMC7491141; doi:10.1186/s13148-020-00934-1)

## Persistent cocaine use EWA

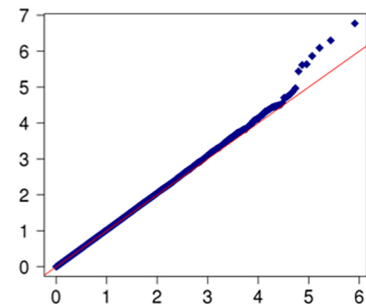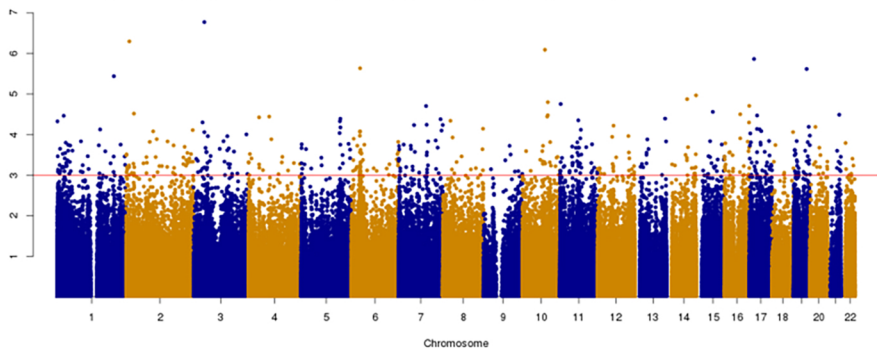

## HIV severity EWA

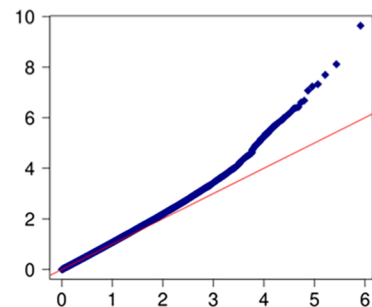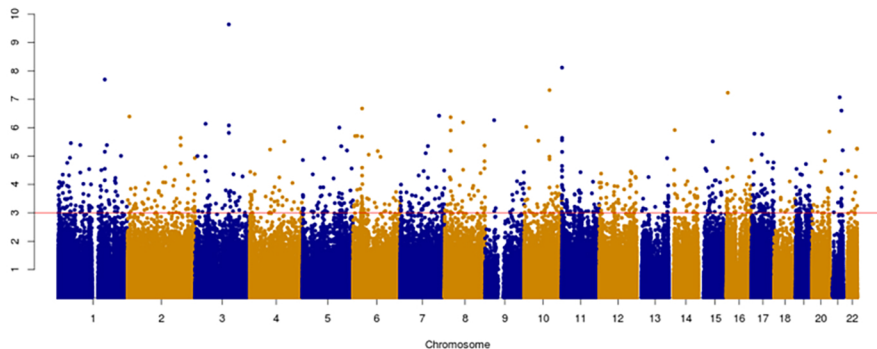

Supplement: Supplementary file 1 — Additional file 1: Figure S1. Manhattan and quantile-quantile (QQ) plot of persistent cocaine use epigenome-wide association (EWA) (λ = 1.034) and HIV severity EWA (λ = 1.116). [file 13148_2020_934_MOESM1_ESM.pdf]

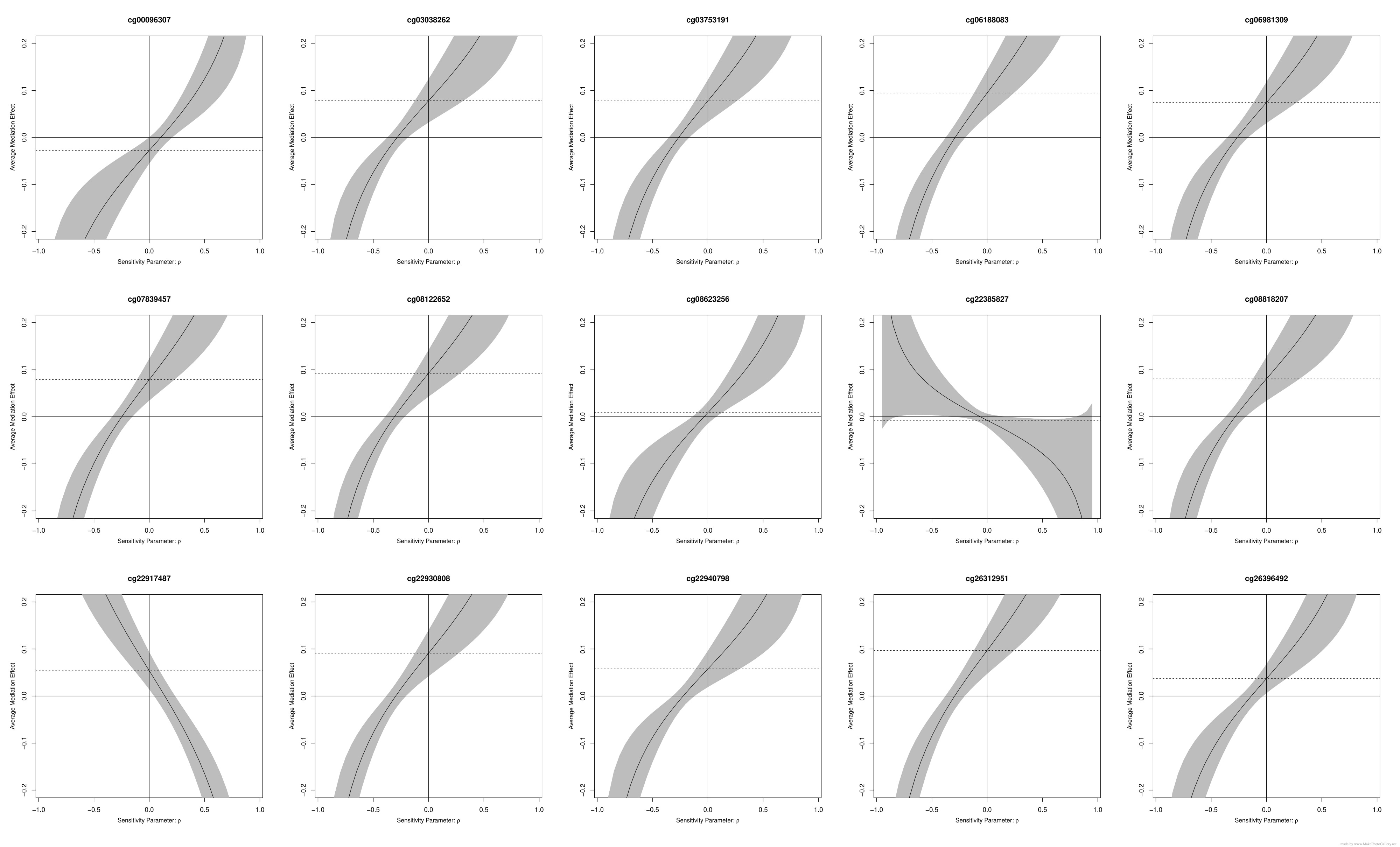

Supplement: Supplementary file 2 — Additional file 2: Figure S2. The estimated ACME and their 95% confidence interval as a function of the sensitivity parameter ρ among 15 candidate CpGs. [file 13148_2020_934_MOESM2_ESM.jpg]
